# From Spot Sign to Bleeding on the Spot: Classic and Original Signs of Expanding Primary Spontaneous Intracerebral Hematoma

**DOI:** 10.1155/2021/9716952

**Published:** 2021-11-15

**Authors:** Ali Kanj, Abir Ayoub, Malak Aljoubaie, Ahmad Kanj, Assaad Mohanna, Feras Chehade, Georges Rouhana

**Affiliations:** ^1^Radiology Department, Hammoud Hospital University Medical Center, Lebanon; ^2^Radiology Department, Lebanese University, Lebanon; ^3^Radiology Department, Beirut Arab University, Lebanon; ^4^American University of Beirut, Lebanon

## Abstract

Expansion of a primary spontaneous intracranial hemorrhage (PSICH) has become lately of increasing interest, especially after the emergence of its early predictors. However, these signs lacked sensitivity and specificity. The flood phenomenon, defined as a drastic increase in the size of a PSICH during the same magnetic resonance study, was first described in this paper based on the data of a university medical center in Lebanon. Moreover, further review of this data resulted in 205 studies with presumed diagnosis of primary spontaneous intracranial hemorrhage within the last 10 years, of which 29 exams showed typical predictors of hematoma expansion on computed tomography. The intended benefit of this observation is to draw the radiologists' attention towards minimal variations in the volume of the hematoma between the two extreme sequences of the same MRI study, in order to detect inconspicuous flood phenomena—a direct sign of hematoma expansion.

## 1. Introduction

Primary spontaneous intracranial hemorrhage (PSICH) refers to any nontraumatic bleeding within the brain parenchyma where no culprit congenital or acquired anomaly could be tracked back. It represents alone around 70-80% of the total number of spontaneous intracranial bleeds [1]. In the vast majority of cases, a fertile, bleeding-prone ground is identified, with association of chronic hypertension and cerebral amyloid angiopathy (CAA) with hemorrhage into the deep brain structures and lobar bleeding, respectively [2]. Despite comprising 10-15% of all strokes, PSICH represents a bigger threat than either ischemic stroke or SAH owing to its high fatality rate [1].

While many clinical and laboratory findings were found to be independent risk factors for poor outcome of intracranial hemorrhage, reliable radiologic signs were limited to the initial volume of the hematoma at presentation and its expansion during the first 6 hours [3]. Hematoma expansion is defined as a relative 33% or absolute 6 ml increase in the initial hematoma volume within the hyperacute phase [4]. Extension of the hematoma into the ventricular system was recently added to this definition raising the prevalence of hematoma expansion to 25.3 and 33.1% of the total number of PSICH [5].

In view of its poor prognosis, anticipation of an expanding hematoma is invariably important to adopt a strict therapeutic approach early in the course of the admission. Many signs were found to predict hematoma expansion on the initial imaging that may vary according to the chosen technique [6].

In this paper, we review classic imaging predictors of expanding hematomas for patients presenting with PSICH to our institution between 2010 and 2020, as well as a first documented case of expanding hematoma on the spot.

## 2. Methods

A thorough review of the radiology department's archive between 2010 and 2020 at Hammoud Hospital University Medical Center (HHUMC) was done, and cases of PSICH were retrospectively identified according to the patients' presentation, characteristic findings on imaging, and surgical reports if any performed. Corresponding NCCTs, CTAs, and MRIs performed urgently upon admission at the emergency department were reexamined by two radiologists with more than 10 years of expertise in this field, for classic predictors of hematoma expansion, as per their definition when first described.

Radiologic studies enrolled in this review belonged to patients with history of uncontrolled hypertension or cerebral amyloid angiopathy, admitted for hypertensive crisis, found to have an intracerebral bleed due to either chronic hypertension or CAA with radiological findings suggestive of hematoma expansion. Of these we mention rapid hematoma expansion on the spot during MRI acquisition (subsequently referred to as the flood phenomenon), ventricular extension of the hematoma, spot sign, blend sign, black hole sign, satellite sign, and island sign. No demographic consideration was taken into account in cases' selection.

Patients with traumatic brain injury, hemorrhagic transformation of an ischemic stroke, subarachnoid hemorrhage, vascular malformation, and intracranial tumoral process have been excluded.

## 3. Results

In the reviewed 10 years, 194 CTs and 11 MRIs showed features of PSICH, with a notable predominance of the older age group among others (>50 years old). Of these, 29 CTs and 1 MRI exhibited signs of impending hematoma expansion. Between the unusual flood phenomenon perceived on MRI and the classic signs detected on CT, findings were divided as follows.

### 3.1. The Flood Phenomenon

#### 3.1.1. Clinical Presentation

A 59-year-old male patient with a known history of hypertension and diabetes was brought to the emergency ward at HHUMC at 00:40 am for acute onset of decreased level of consciousness and elevated blood pressure that started within 30 minutes prior to presentation. At admission, his Glasgow Coma Score (GCS) was 5, blood pressure 250/110 mmHg with concomitant respiratory acidosis, for which he was intubated. ECG and laboratory tests were unremarkable. Urgent MRI brain was done, starting at 1:05 am.

#### 3.1.2. Radiological Findings

The patient was scanned by a GE 3T discovery 750W, using consecutively the following sequences: DWI, FLAIR, axial T2WI, susceptibility-weighted angiography (SWAN), FSE, and 3D TOF.

**Figure 1:**
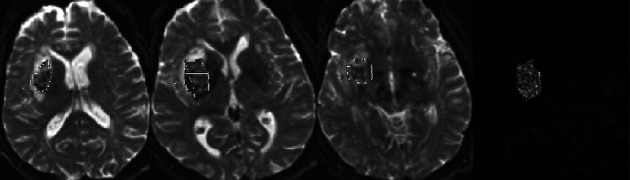
T2WI axial section showing hemorrhage segmentation at the beginning of the examination (red outline), with a 3D reconstruction at the same stage.

**Figure 2:**
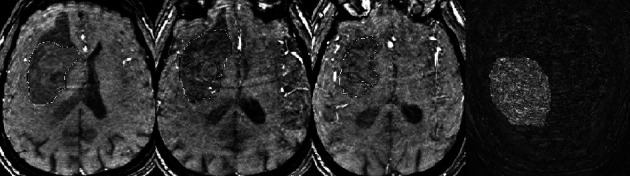
GRE axial section showing hematoma segmentation at the end of the examination and a 3D reconstruction of the hematoma with an obvious increase in its size referred to as the flood phenomenon.

The retrospective evaluation of these sequences in the same sequential order showed a focal active hemorrhagic lesion at the level of the right central gray matter mainly involving the globus pallidus and putamen. The size of the hematoma was assessed using the thresholding method with image segmentation on axial sequences and volume rendering. A striking increase in the size of the hematoma between the native and the final sequences was noted, starting with 8.1 ml (3.2 × 3.2 × 1.6 cm) on the DWI (Figure 1) and reaching 56.6 ml (6 × 5.9 × 3.2 cm) on the 3D TOF exerting a significant mass effect over the right lateral ventricle (Figure 2). These findings are compatible with an active, on spot, bleeding caused by the rapidly flowing blood from many small capillaries into an impervious ground (central gray matter), mimicking the naturally occurring flood phenomenon.

The lack of a nidus or mass and the characteristic location of the bleed favored the diagnosis of PSICH over any alternative diagnosis.

#### 3.1.3. Management and Outcome

The patient was started on nicardipine syringe pump for aggressive control of the systolic blood pressure (<160 mmHg and <140 mmHg at best) and underwent craniotomy for hematoma evacuation within the first 12 hours of presentation. However, clinical and hemodynamic status kept deteriorating with EEG flattening on the second day post op and subsequent cardiac arrest in the following day.

### 3.2. Classic Signs of Expanding Hematoma

The remaining 29 CT scan-based predictors of hematoma expansion were distributed as follows. The spot sign was observed in 4 injected CT scans, and the black hole sign and the blend sign were seen in 6 and 3 nonenhanced studies, respectively, while the island sign was documented in 4 and the satellite sign in 3 out of the 29 scanners regardless of the enhancement. In 14 CT scans, the hematoma extended towards the ventricles. This sign was isolated in 9 cases and was associated to the island sign in 3 cases, the spot sign in 1 case, and the black hole sign in 1 case as well.

**Figure 3:**
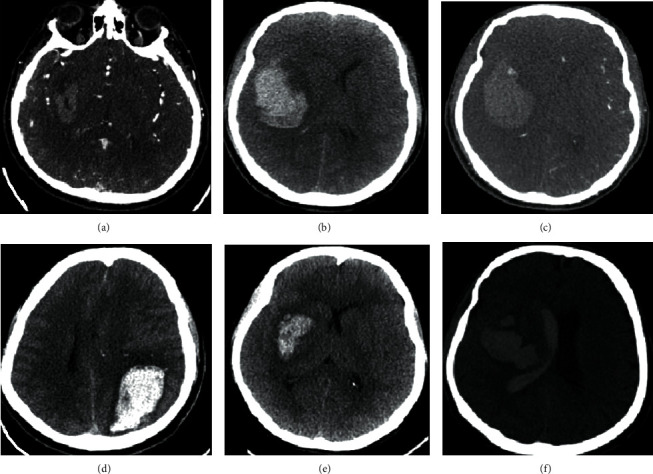
Signs of hematoma heterogeneity predicting expansion: (a) the “black hole” sign, (b) the “blend” sign, (c) the “spot” sign, (d) the “island” sign, (e) the “satellite” sign, and (f) a parenchymal hematoma extending into the lateral ventricle.

Further details and pictures are found in Figure 3.

## 4. Discussion

Hematoma expansion, despite being an independent risk factor of poor prognosis in PSICH, is nevertheless a modifiable one, whose early prediction could lead to a better anticipation of complications and selection of high-risk groups in whom vigorous measures are needed. Owing to its availability, speed, and cost effectiveness, CT has been considered the test of choice for initial diagnosis of PSICH. Its implementation has led to both a rapid diagnosis of bleeding and reliable predictors of hematoma expansion in these settings.

During the last two decades, innumerable studies were conducted in this filed and a substantial haul of signs could be collected from the literature (Table 1).

Of these, the size of the initial hematoma showed the greatest correlation with the risk of its expansion. The sensitivity and specificity of this finding were the highest ever recorded of 96% and 98%, respectively [3], followed by margin irregularity (sensitivity 70%, specificity 46%) [7]. Furthermore, other typical patterns of the heterogeneous density within the hematoma are recognized. Being the sign of the greatest interest, the spot sign recorded a sensitivity between 51 and 62% with a specificity reaching 88% for progression of the bleeding, neurologic deterioration, and death.

Other indirect signs of which the blend sign, swirl sign, black hole sign, island sign, and satellite sign showed an encouraging specificity reaching 94% in its lowest, but a disappointing sensitivity of 40% at best [6].

Recently, MRI was added to the initial imaging modalities in use for the diagnosis of PSICH, proving a comparable sensitivity to CT and a superior specificity for detecting underlying vascular conditions. However, it is still rarely done within the acute phase.

A callout to overcome the shyness of using MRI in the settings of an acute PSICH was made by Christine et al. when it was found that MRI has changed the presumptive diagnosis made on CT and therefore the management, in no less than 22% of cases [8].

Since its first description by Murai et al. [8, 9], records of spot sign on MRI were limited to 4 papers with small sample size, showing a sensitivity of 36 to 40% for bleeding on SWI and GRE, good correlation with contrast extravasation on CT, and a statistically significant association with poor prognosis [10].

The closest the MRI acquisition has ever been to the onset of the bleeding was after 23 minutes of the latter, reported in a study by Linfante et al. [11] who gathered 5 MRIs done within 2 hours from the last known normal neurologic state of the patients presenting with PSICH.

Characteristic features of the intracerebral hemorrhage in the hyperacute phase were noted on SWI, dividing the lesion into 3 zones: the central hyper/isointense area (proteinaceous fresh blood), the peripheral hypointense area (deoxygenated hemoglobin), and the surrounding hyperintense rim (vasogenic edema).

The relatively long time required for MRI acquisition has always been regarded as the major drawback of this technique, despite the precise diagnosis it provides. This paper, aside from reviewing some of the previously reported predictors of a growing hematoma, points out the other edge of this sword. The additional time spent in the magnet did not only allow the manifestation of a probable predictor of hematoma expansion, rather it exhibited an irrefutable evidence of the latter, with a striking 4 times increase in the initial hematoma volume during the same exam.

To the best of our knowledge, this is the first reported case of expansion of an intracranial hematoma during the same radiological study, giving a real-time observation of the changes within the hematoma during the active bleeding. This gave the impression of a flood phenomenon, because as in a flash flood, a high velocity torrent of blood occurred in a susceptible area with a deleterious outcome. This incident is exceptional, particularly in a SICH where the source of the streaming blood is merely some small vessels with a slow flow. However, it could be attributed to the chronic severe damage affecting a wide area of the cerebral vascular bed struck by the prolonged episode of extremely high blood pressure leading to higher volumes of afferent blood and rapid hematoma expansion.

Such observation could not be possible if any other rapid single-scan technique has been used. If adequately interpreted, it can represent the Newton's apple for less noticeable floods of more frequent occurrence, allowing to objectively foresee further hematoma expansion based on smaller differences between the volume of the intracranial hematoma in the first and last MRI sequences taken during the same examination, theoretically proportional to the risk of further aggravation.

However, our review did not collect a sufficient number of MRI studies to accurately assess the applicability and usefulness of this method or to precise the minimal discrepancy required and the appropriate range of errors. Therefore, additional studies with large sample size are needed.

## Figures and Tables

**Table 1:** Signs of hematoma heterogeneity or in predicting its expansion.

Signs	Imaging modality	Author	Definition
Flood phenomenon	MRI	First reported in the current article	Increase in the size of the intracranial hematoma between the first and last MRI sequences during the same examination
Margin irregularity	NCCT or CTA	Blacquiere et al. [7]	Indicates bleeding multifocality
Mean CT attenuation value	NCCT	Chu et al. [12]	If <31 UH, it indicates an impaired coagulation and higher risk of expansion
Extension into the ventricles	NCCT or CTA	Deng et al. [13]	The parenchymal bleed dissects into the ventricles
Spot sign	Enhanced CT	Demchuk et al. [4]	>1.5 mm dot-like appearance without connection to an outside vessel or corresponding density on NCCT twice as dense as the remaining hematoma
Blend sign	NCCT	Li et al. [14]	The mixture of the fresh central hyperdense blood with the older peripheral blood
Black hole sign	NCCT or CTA	Li et al. [15]	Central hypodense streaming blood encapsulated within the hyperdense coagulating hematoma (minimum of 28 HU difference between both)
Island sign	NCCT or CTA	Li et al. [16]	Either at least 3 small hematomas totally separate from the main focus or 4 small foci that may bud from the initial hematoma
Satellite sign	NCCT or CTA	Shimoda et al. [17]	Single small hemorrhage < 1 cm, completely isolated from the initial bleed
Swirl sign	NCCT	Boulouis et al. [18]	Hypodense area found on 2 consecutive 5 mm axial CT slices
Leakage sign	Contrast-enhanced CT between arterial and delayed phase	Koculym et al. [19]	A 10% increase in the density of the hematoma over a region of interest of 1 cm diameter between the arterial and the delayed phase

## Data Availability

Data are available on request.

## Abbreviations

CAA: Cerebral amyloid angiopathy
GCS: Glasgow Coma Score
HHUMC: Hammoud Hospital University Medical Center
HU: Hounsfield unit
PSICH: Primary spontaneous intracranial hemorrhage
SWAN: Susceptibility-weighted angiography.
